# Enhanced ResU-Net for brain tumor segmentation using EfficientNetB0, channel attention, and ASPP

**DOI:** 10.1088/2057-1976/ae6459

**Published:** 2026-05-05

**Authors:** Majid Behzadpour, Ebrahim Azizi, Bengie L Ortiz, Kai Wu

**Affiliations:** 1 Department of Electrical and Computer Engineering, Texas Tech University, Lubbock, TX 79409, United States of America; 2 Department of Rehabilitation and Movement Sciences, School of Health Professions, Rutgers Health, Newark, NJ 07107, United States of America

**Keywords:** deep learning, brain cancer, MRI images, computer-aided diagnosis, tumor segmentation

## Abstract

Accurate and efficient segmentation of brain tumors is critical for diagnosis, treatment planning, and monitoring in clinical practice. In this study, we present an enhanced ResU-Net architecture for automatic brain tumor segmentation, integrating an EfficientNetB0 encoder, a channel attention mechanism, and an atrous spatial pyramid pooling (ASPP) module. The EfficientNetB0 encoder leverages pre-trained features to improve feature extraction efficiency, while the channel attention mechanism enhances the model’s focus on tumor-relevant features. ASPP enables multi-scale contextual learning, which is crucial for handling tumors of varying sizes and shapes. The proposed model was evaluated on two benchmark datasets: The Cancer Genome Atlas Low Grade Glioma and brain tumor segmentation (BraTS-2020). Experimental results demonstrate that our method consistently outperforms the baseline ResU-Net and its EfficientNet variant, achieving dice similarity coefficient of 0.903 and 0.851, and HD95 scores of 9.43 and 3.54 for whole tumor and tumor core (TC) regions on the BraTS 2020 dataset, respectively. Compared to state-of-the-art methods, our approach shows competitive performance, particularly in whole tumor and TC segmentation. These results indicate that combining a powerful encoder with attention mechanisms and ASPP can significantly enhance BraTS performance. The proposed approach holds promise for further optimization and application in other medical image segmentation tasks.

## 1. Introduction

Brain tumors arise as a result of the uncontrollable proliferation of abnormal cells. Although their exact causes are not well-known, some of the risk factors include family history, metastases, and exposure to ionizing radiation [1]. A very common type of brain tumor is glioma. The World Health Organization has divided glioma into two categories: low-grade (Grade I and Grade II) gliomas and high-grade (Grade III and Grade IV) gliomas. Low-grade gliomas (LGG) tend to be less aggressive, with longer survival rates, while high-grade gliomas (HGG) exhibit rapid progression and require urgent medical intervention. The median survival rate for HGG is less than two years, with observed survival rates of approximately 42.4% at six months, 17.7% at one year, and 3.3% at two years [2, 3].

Magnetic resonance imaging (MRI) is a primary modality for brain tumor diagnosis due to its non-invasive nature and high-resolution imaging capabilities, providing detailed structural, functional, and diffusion information [4]. Accurate tumor segmentation from MRI is essential for diagnosis, treatment planning, and monitoring. However, manual segmentation remains time-consuming and subject to inter- and intra-observer variability [1, 5], motivating the development of automated approaches. The challenge in segmenting brain tumors lies in the differences in shape, size, and location that each tumor assumes from one patient to another. Most HGG invade the surrounding normal-appearing tissues; hence, their borders cannot be well delineated. Additionally, MRI images obtained from various modalities such as flair, T1, contrast-enhanced T1, and T2 differ in their degree of contrast, making segmentation even more challenging [6, 7]. Traditional machine learning techniques relied on manual feature extraction and labeled datasets for training classifiers; however, these usually faced ambiguous boundaries between malignant and benign tissues and needed substantial expert input at the initial annotation stage [6].

Recent advancements in deep learning have revolutionized brain tumor segmentation (BraTS). Convolutional neural networks (CNNs) and fully convolutional networks (FCNs) have demonstrated remarkable success in medical image analysis due to their ability to learn complex hierarchical features from large datasets [8, 9]. Notably, the introduction of the BraTS challenge (BraTS) has accelerated the development of robust, standardized models for BraTS by providing high-quality annotated datasets [10]. U-Net and its variants have become the most widely adopted deep models in BraTS, often achieving state-of-the-art performance compared to other deep learning-based models [11]. However, such networks usually have a few limitations: they are not effective at modeling complex contextual features or processing multi-scale information efficiently.

In this work, we propose an improved ResU-Net architecture for BraTS using multimodal MRI data. Our model is based on some previous works in ResU-Net-based architectures and embodies several key improvements to enhance the accuracy of segmentations:

• Channel attention mechanisms: the traditional U-Net model regards all channels with equal importance, so we will use a channel attention mechanism. This approach enables the network to selectively emphasize the most informative channels of each MRI modality for enhancing the focus on tumor regions.
• Atrous spatial pyramid pooling (ASPP): an ASPP module is integrated at the bottleneck to capture multi-scale contextual information, which is quite essential for the accurate segmentation of tumors with varied sizes and shapes. ASPP lets the model embed both local and global contextual clues [12].

We further enhance our approach by leveraging EfficientNet as the backbone encoder, which provides a powerful feature extraction capability while maintaining computational efficiency [13]. Its compound scaling strategy ensures a balanced trade-off between model depth, width, and resolution, allowing for better representation of complex brain tumor features with reduced computational costs. We evaluate our model on two benchmark datasets: the Cancer Genome Atlas LGG (TCGA-LGG) dataset, containing 3929 MRI scans of 110 patients, and BraTS2020, including multimodal MRI scans from 369 patients [14–16]. The TCGA dataset would better represent real-world variations, while BraTS2020 includes standardized ground truth annotations and is, therefore, more suitable for benchmarking segmentation models. Our approach targets state-of-the-art performance by addressing the key limitations of existing models through advanced architectural modifications.

## 2. Related works

The field of deep learning models for BraTS has undergone extensive exploration. Deep neural networks have recently become the favored choice, dominating most works on BraTS, particularly since larger datasets have been beneficial to them. Kamnitsas *et al* proposed an ensemble approach, combining predictions from multiple 3D convolutional networks, including DeepMedic, FCN, and U-Net [17–19]. Similarly, Myronenko investigated a cascaded U-Net model, where a coarse segmentation obtained from the first stage was further refined by a second-stage U-Net [20]. Isensee *et al* applied nnU-Net, a self-configuring method that adapts U-Net to particular datasets, achieving state-of-the-art performance in the BraTS challenge [21]. Further variants of nnU-Net, including the use of group normalization instead of batch normalization and the addition of axial attention, have presented higher segmentation accuracy [22].

The attention mechanism has played a pivotal role in improving BraTS. Noori *et al* applied channel attention immediately after the concatenation of low-level and high-level features in a 2D encoder–decoder architecture, emphasizing the importance of weighing different features over naive concatenation [23]. Zhang *et al* introduced attention gates at the skip connections, further improving segmentation accuracy [24]. Additionally, Cao *et al* proposed a U-Net-like model using 3D shuffle attention in the encoder for better feature extraction and skip connections [25]. These studies highlight how the inclusion of attention mechanisms enhances segmentation quality.

U-Net is one of the foundational architectures for segmenting medical images. The model proposed by Ronneberger *et al* includes a contracting path for feature extraction and an expansive path for precise localization through upsampling [26]. The concatenation of encoder and decoder features through skip connections enhances segmentation accuracy by preserving spatial details. Furthermore, hybrid models, such as U-Net combined with other architectures like ResNet, have achieved excellent results in BraTS tasks, with dice similarity coefficients (DSCs) higher than 0.90 on benchmark datasets [26–28]. Recent works also feature a genetic algorithm-based CNN that automatically optimizes the network architecture, achieving accuracy up to 0.94 on the TCGA dataset [29].

Further developments in BraTS involve modifications of the standard U-Net model for better performance. Baid *et al* presented a 3D patch-based U-Net for glioma segmentation and survival prediction [30, 31]. Their approach, using a deep learning radiomics algorithm for gliomas, achieved significant results, with DSC values of 0.9795 for HGG and 0.9950 for low-grade gliomas. Another noteworthy approach is the spatial pyramid pooling U-Net (SPP-U-Net), which integrates SPP with attention blocks to improve multi-scale feature representation [32]. Additionally, researchers like Roy *et al* have introduced advanced architectures, such as S-Net and SA-Net, that utilize attention-based mechanisms to enhance segmentation results on MRI scans [33].

Recent advancements also explore new architectures beyond traditional CNNs. For instance, Ruba *et al* proposed JGate-AttResU-Net, which incorporates an attention gate and residual blocks to reliably highlight variably sized tumor regions [34]. Yanjun Peng and Jindong Sun proposed an efficient automatic weighted dilated convolutional network (AD-Net) architecture, based on weighted dilated convolutions and deep supervision, to enhance segmentation efficiency [35]. More recently, transformer-based models like TransBTS have shown that attention-driven models are capable of learning robust features for BraTS [36]. These works highlight the trend of using more complex neural network elements, including transformers and multi-scale feature fusion, to achieve better segmentation results.

Recent studies have explored MRI-based radiomics approaches for non-invasive tumor characterization and clinical outcome prediction. For instance, an XGBoost-based model predicted 1*p*/19*q* co-deletion status in low-grade glioma patients using optimized imaging features, achieving 82.8% accuracy on an external test set and reducing reliance on invasive procedures [37]. Similarly, another study developed an MRI-based radiomics model for survival prediction, reporting strong performance with an average iAUC of 0.867 on the test set across both GBM and LGG dataset [38]. These works highlight the growing role of imaging-derived features and machine learning in improving clinical decision-making for brain tumor analysis.

Despite these advancements, many existing methods rely on computationally intensive 3D architectures or lack a unified integration of multi-scale contextual learning and adaptive feature refinement. This limits their efficiency and practical applicability. To address these limitations, we propose an enhanced ResU-Net framework that combines an EfficientNet encoder, channel attention, and ASPP for improved feature representation and multi-scale context modeling.

## 3. Methodology

**Figure 1.**
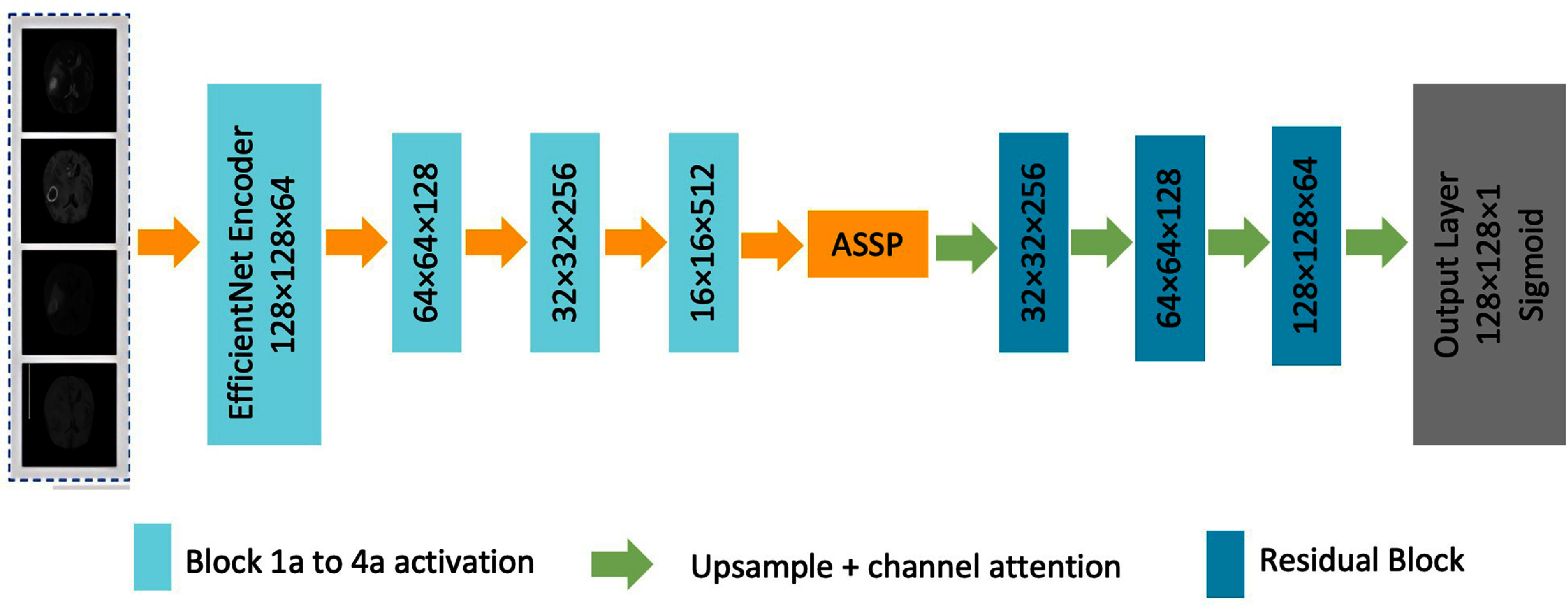
The architecture of the proposed 2D ResU-Net-based model, leveraging an EfficientNet encoder for feature extraction, followed by multi-scale feature fusion in the bottleneck, and a decoder comprising an attention block, residual connections, and upsampling layers.

In this work, we propose an improved version of the ResU-Net model as the backbone of our approach to BraTS. First, we propose the structure of ResU-Net, enhanced by the EfficientNetB0 encoder, to leverage pre-trained features and improve efficiency. Then, we introduce the channel attention mechanism, showing how it serves to help in feature selection by emphasizing the relevant regions, ensuring the model focuses on critical tumor areas. Next, we incorporate ASPP in the bottleneck to model multi-scale contextual information, enhancing the model to recognize tumors of varying sizes. The integration of residual blocks for facilitating better gradient flow and refinement is retained, mitigating vanishing gradient issues and enhancing learning stability. Figure 1 shows the components of the proposed ResU-Net-based model.

### 3.1. Baseline model: ResU-Net with EfficientNetB0 encoder

Our baseline model relies on the ResU-Net architecture [39], incorporating an EfficientNetB0 encoder [13]. The network has an encoder–decoder structure with skip connections between corresponding layers. The multi-modal MRI inputs are processed by a pre-trained EfficientNetB0 backbone initialized with ImageNet weights that can be further fine-tuned during training [40]. EfficientNetB0 was chosen for its superior feature extraction capability with fewer parameters, enabled by its compound scaling strategy, which balances network depth, width, and resolution efficiently, achieving higher DSC while maintaining lower computational costs [41].

The encoder pathway utilizes four key activation layers from EfficientNetB0 (block1a through block4a) to extract hierarchical features. The pathway in the decoder comprises a total of four upsampling levels, reducing feature channels from 256 to 32 through transposed convolutions with a stride of 2. At each level, the decoder receives skip connections from the corresponding encoder activation layer, followed by a residual block for feature refinement [27].

This final output layer consists of a 2 × 2 transposed convolution that upsamples to the input resolution, followed by a 1 × 1 convolution with sigmoid activation to generate three output channels corresponding to the whole tumor, tumor core (TC), and enhancing tumor regions.

### 3.2. Channel attention mechanism

Our network incorporates channel attention mechanisms in the decoder pathway to adaptively recalibrate channel-wise feature responses. The channel attention mechanism emphasizes informative feature channels while suppressing less important ones. The attention module is applied after each skip connection concatenation in the decoder pathway to effectively calibrate the merged features from different scales. Channel attention has been shown to improve segmentation accuracy through enhanced feature discrimination and reduction in irrelevant activations, leading to more robust and precise predictions [42].

Given an input feature map *F ∈ ℝ^(H × W × C)*, the channel attention module first generates two different channel descriptors through global average pooling ${F_{{\mathrm{avg}}}}$ and global max pooling ${F_{{\mathrm{max}}}}$ operations:

\begin{equation*}{F_{{\mathrm{avg}}}} = \frac{1}{{H \times W}}\mathop {\mathop \sum\nolimits }\limits^H_{i = 1} \mathop {\mathop \sum \nolimits }\limits^W_{j = 1} F\left( {i,j} \right)\end{equation*}

\begin{equation*}{F_{{\mathrm{max}}}} = \mathop {\max }\limits_{i,j} F\left( {i,j} \right)\end{equation*}

where *F(i,j)* represents the feature value at spatial position *(i,j)*. The channel attention map ${M_{\mathrm{c}}}$ is then computed by:

\begin{equation*}{M_c} = {\text{ }}\sigma \left({F_{\mathrm{avg}}} + {F_{\max }}\right)\end{equation*}

where *σ* denotes the sigmoid activation function. The final output $F^{\prime}$ is obtained by rescaling the input feature map with the attention weights:

\begin{equation*}F^{\prime} = {M_{\mathrm{c}}} \otimes F,\end{equation*}

where ⊗ denotes channel-wise multiplication. The attention mechanism is systematically integrated at four levels in the decoder pathway, processing feature maps with varying channel depths (256, 128, 64, and 32 channels). This hierarchical application of channel attention helps in refining features at multiple scales, particularly after the fusion of skip connections with upsampled features, leading to more discriminative feature representations for accurate tumor segmentation.

### 3.3. ASPP

In particular, we embed an ASPP module at the bottleneck of the network to capture multi-scale contextual information that is critical for segmenting brain tumors of diverse sizes and shapes [43]. This ASPP module performs parallel atrous convolutions at different dilation rates of 6, 12, and 18, along with a 1 × 1 convolution branch and a global pooling branch. The ASPP allows the model to capture both fine-grained and high-level semantic information by using multiple dilations, making it effective in capturing tumors of varying sizes and irregular shapes. Each branch processes the input features using 256 filters, followed by ReLU activation. Features from all the branches are then concatenated and processed through a final 1 × 1 convolution, hence letting the network capture contextual information effectively at different scales with no loss in spatial resolution.

## 4. Experiments

### 4.1. Datasets and augmentation

We utilized two datasets: the BraTS-Glioma dataset provided by the BraTS Challenge 2023 and the TCGA LGG dataset, containing 3929 MRI scans of 110 patients [16]. The BraTS MRI scans in the BraTS-Glioma comprise native (T1), post-contrast T1-weighted (T1Gd), T2-weighted (T2), and T2 fluid attenuated inversion recovery (T2-FLAIR). The primary evaluations have been performed for three tumor sub-regions of interest: the ‘enhancing tumor’ with hyper-intensity in T1Gd—ET, the ‘TC’ that includes both necrotic regions and ET—TC, and the ‘whole tumor’ that includes TC and peritumoral invaded tissue—WT. These datasets were chosen due to their extensive use in BraTS research and their well-annotated multi-modal MRI scans, making them ideal for training and evaluation. The BraTS dataset is widely recognized for benchmarking tumor segmentation models, ensuring comparability with state-of-the-art methods.

Since the BraTS dataset consists of 3D MRI scans, we process the data using a slice-by-slice approach, where each 2D slice is treated as an independent input while maintaining spatial consistency through standardized preprocessing. Each slice is represented as a multi-channel input, incorporating four MRI modalities (T1, T1Gd, T2, and FLAIR) to provide rich contextual information. This study follows a similar design rationale to nnU-Net, emphasizing that systematic architectural optimization and well-designed training strategies can yield competitive performance, even within a 2D framework [44].

From the BraTS data, we used 369 cases for training, 125 cases for validation, and 166 cases for test set (a ratio of 56% for training, 19% for validation, and 25% for testing). From the TCGA-LGG dataset, we adopted 120 cases for training, 28 cases for validation, and 28 cases for the test set (a ratio of 70% for training, 15% for validation, and 15% for testing). Models are trained using many augmentation techniques to increase robustness and prevent overfitting by using random rotation of ±25 degrees that can capture different types of invariances or orientation variability. It includes using random horizontal/vertical shifts that are within 20% of the dimensions of an image, random zoom transformations with ±20% limits, random horizontal flips, and interpolating newly created pixels with one of the following techniques during the above operations: nearest neighbors. These augmentation parameters are carefully selected based on empirical studies and prior works in medical image analysis [45].

All dataset partitions were performed at the patient level to prevent data leakage between training, validation, and test sets, ensuring that slices from the same patient do not appear across different subsets and maintaining a fair evaluation protocol.

### 4.2. Evaluation metrics

To evaluate the performance of our BraTS models, two common metrics were adopted: the DSC and the 95th percentile Hausdorff Distance (HD95), both widely used in medical image analysis. DSC refers to the ratio of overlap between the predicted tumor area and the ground truth annotation; it as:

\begin{equation*}{\mathrm{DSC}} = \frac{{2\left| {P \cap G} \right|}}{{\left| P \right| + \left| G \right|}}\end{equation*}

where ∣*P*∣ and ∣*G*∣ denote the number of pixels in the predicted and ground truth regions, respectively. A higher DSC indicates a better overlap between the real and predicted masks of tumors.

Complementing the DSC, we also use the HD95, which is the robust version of the HD95, computing the 95th percentile of the maximum surface-to-surface distance between predicted segmentation and ground truth boundary. This metric provides the accuracy of the boundary localization and is insensitive to extreme outliers by excluding the worst 5% of boundary mismatches. The mathematical formulation of HD95 is given as:

\begin{equation*}{\mathrm{H}}{{\mathrm{D}}_{95}}\left( {A,B} \right) = {P_{95}}\left( {{\mathrm{max}}\left( {\mathop {\sup }\limits_{a \in A} d\left( {a,B} \right),\mathop {\sup }\limits_{b \in B} d\left( {b,A} \right)} \right)} \right),\end{equation*}

where *d(a,B)* represents the shortest Euclidean distance from a point *a* in segmentation *A* to the closest point in ground truth *B*.

These two metrics, the DSC and HD95, provide a comprehensive measure of volumetric overlap and boundary precision of the segmented tumor regions. However, despite their strengths, they also have limitations. DSC, while effective for measuring overlap, may not fully reflect boundary alignment errors, especially in cases with thin or irregular tumor structures. Similarly, HD95, though robust to extreme outliers, may still be influenced by segmentation inconsistencies in complex tumor shapes. Therefore, interpreting results using both metrics ensures a more balanced evaluation of segmentation accuracy.

### 4.3. Results and discussion

In this section, we present a detailed analysis and comparison of the quantitative results obtained from the baseline ResU-Net, Res-UNet with EfficientNet backbone, and our proposed enhanced method incorporating channel attention and ASPP mechanisms. The evaluation was performed on two distinct datasets: TCGA LGG and BraTS 2020.

The proposed method demonstrates potential for clinical applicability by achieving consistent improvements in tumor boundary delineation across two publicly available datasets, which is essential for reliable treatment planning and longitudinal monitoring. Our model emphasizes a balance between segmentation accuracy and computational efficiency by leveraging an EfficientNet encoder and channel attention mechanisms for multi-scale feature. While external validation on independent institutional datasets and domain-shift analysis is beyond the scope of this study, the use of two heterogeneous benchmarks (TCGA LGG and BraTS 2020) provides initial evidence of generalization capability. In addition, the 2D design enables relatively efficient inference with a lower computational burden compared to many transformer-based models, supporting its feasibility for integration into clinical workflows. Future work will focus on validating the model in real-world clinical environments, including multi-center data evaluation and analysis of its impact on downstream tasks such as prognosis estimation and treatment decision support. Furthermore, the model was trained using k-fold cross-validation on the training set to enhance robustness and reduce sensitivity to data variability. This strategy allows the model to be exposed to different subsets of the data during training, improving its generalization capability and stability. The final evaluation was conducted on independent test sets to ensure an unbiased assessment of performance.

#### 4.3.1. Comparison of the proposed method

Quantitative results. Tables 1 and 2 show the comparisons of our proposed method with the baseline approaches. The proposed improved model consistently outperforms the different variations of baseline methods for both datasets. On the TCGA LGG dataset, the results indicate that our proposed method provides the top segmentation performance with a DSC of 0.797 and HD95 of 6.16, which is considerably improved from the baseline Res-UNet (DSC: 0.787, HD95: 7.21).

**Table 1.** Compared segmentation results with baselines on the TCGA LGG test dataset.

Methods	Aug	DSC	HD95
Res-Unet		0.787	7.21
Res-Unet	✓	0.773	7.29
Res-Unet + EfficientNet		0.775	6.47
Res-Unet + EfficientNet	✓	0.781	6.39
Our Method	✓	**0.797**	**6.16**

**Table 2.** Compared segmentation results with baselines on the BraTS2020 test dataset.

		DSC			HD95		
Methods	Aug	ET	WT	TC	ET	WT	TC
ResU-Net		0.722	0.851	0.767	7.20	11.65	5.54
ResU-Net	✓	0.725	0.863	0.770	7.18	11.13	6.21
ResU-Net + EfficientNet		0.741	0.877	0.781	6.25	10.41	5.34
ResU-Net + EfficientNet	✓	0.750	0.889	0.789	6.35	10.08	5.14
Our method	✓	**0.762**	**0.903**	**0.794**	**5.87**	**9.43**	**3.54**

The baseline ResU-Net struggles with boundary refinement and lacks effective feature recalibration, leading to higher HD95 values. Its combination with the EfficientNet backbone and data augmentation demonstrated a mediocre boost: (DSC: 0.781, HD95: 6.39), suggesting that while a stronger encoder enhances feature extraction, it alone does not fully optimize segmentation performance, whereas our final method further improved the segmentation results by including channel attention and ASPP. However, despite these improvements, certain challenges remain. First, small and disorganized tumors are difficult to segment with loss of spatial information in downsampling operations, with fine-grained information not recovered well through skip operations. Receptive field constraints in early layers make it difficult to detect faint areas of a tumor, and bias in Dice loss towards larger structures lessens vulnerability to small lesions. Second, boundary refinement is still limited, as feature smoothing in deep layers and restrictions in skip operations contribute to over-smooth prediction and failure of the model-to-model sharp transition and indeterminate borders of a tumor accurately. Despite improvement in multi-scale feature extraction using ASPP, it attends less to boundary information and sharp detail in general. Third, modality variation in multi-modal MRI introduces generalizability concerns through intensity and contrast variation between scanners, which channel attention alone cannot fully address. Where data augmentation helps mitigate domain shifts, it cannot eliminate all variations in tumor appearance between protocols.

On the BraTS 2020 dataset, the proposed approach reports the best performance in the three tumor regions: ET, WT, and TC. The improved model achieves DSC of 0.762, 0.903, and 0.851 for ET, WT, and TC, respectively, significantly outperforming both a baseline ResU-Net and its EfficientNet variant. HD95 also improved significantly, most notably in the TC region, where our approach achieved 3.54 against the baseline value of 5.54. The most difficult part for segmentation, the whole tumor area, demonstrates a much-improved DSC (0.903) and low HD95 of 9.43 against the baseline of 0.851 and 11.65, respectively.

A *p*-value threshold of *p* < 0.05 was set to define statistical significance in the Wilcoxon signed-rank test. On the TCGA LGG dataset, the improvement in DSC from 0.781 to 0.797 was found to be statistically significant, *p* = 0.012, and the reduction in HD95 (from 6.39 to 6.16) also yielded a significant result, *p* = 0.003. For the BraTS 2020 dataset, the performance leap in the TC region, where HD95 dropped from 5.14 to 3.54, produced a highly significant *p*-value of *p* < 0.001. These results confirm that the integration of channel attention and ASPP mechanisms provides a robust and non-random enhancement to the segmentation architecture.

The impact of data augmentation varies across different model configurations. While the baseline ResU-Net shows mixed results with augmentation (slight decrease in TCGA LGG performance but improvement in BraTS 2020), the EfficientNet backbone consistently improved from augmentation across both datasets. This could indicate that the more complex architecture is better at using the augmented data in order to learn robust features.

**Figure 2.**
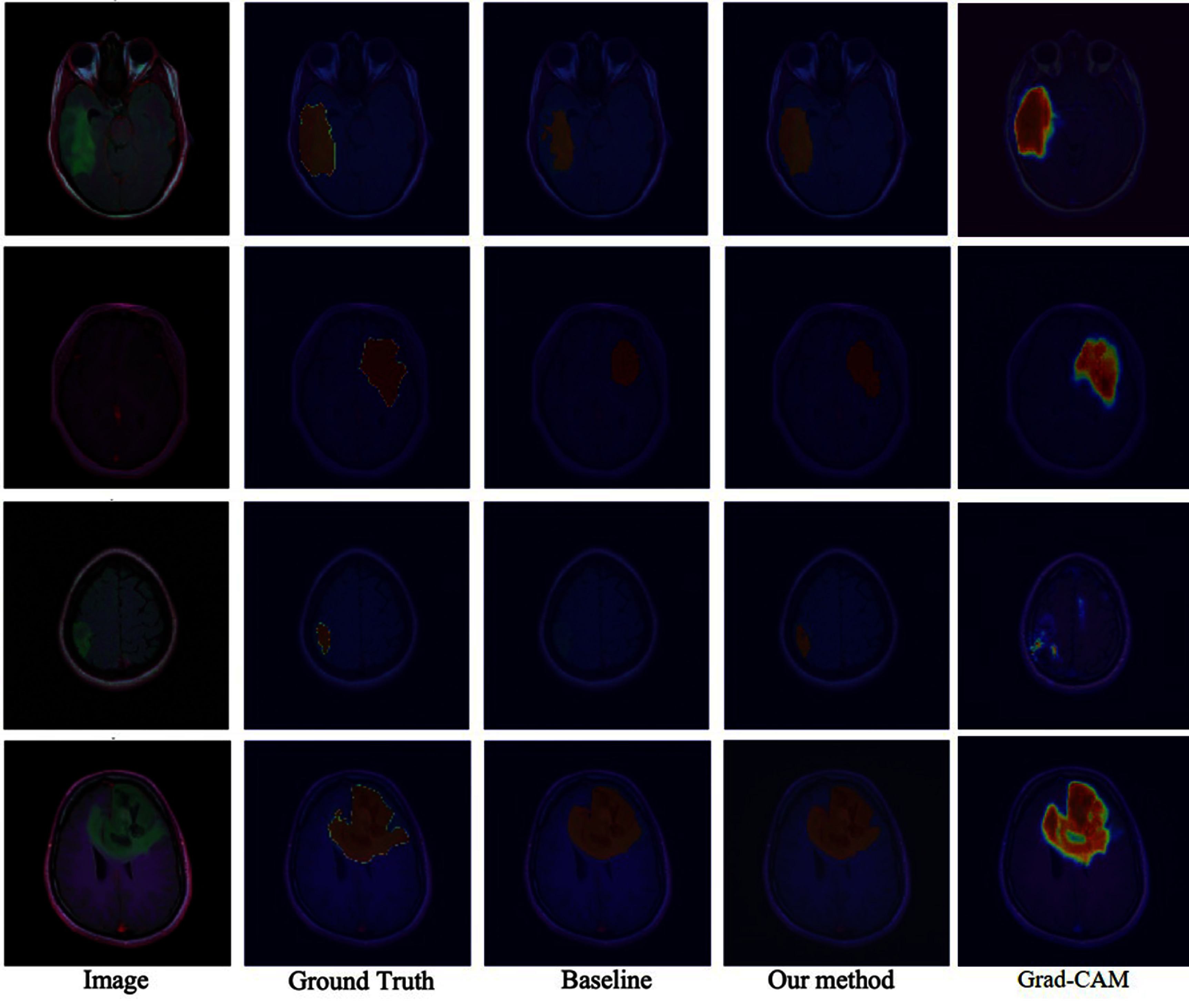
Segmentation results provided by the standard ResU-Net and our method. This result was obtained from the test set. Reproduced with permission from [15].

Figure 2 shows representative segmentation results comparing our proposed method with the baseline approaches. The visual comparison demonstrates that our enhanced model, incorporating channel attention and ASPP modules, produces more precise tumor boundaries that closely align with the ground truth, particularly in areas where the baseline ResU-Net struggles to maintain boundary consistency. This visual improvement validates the quantitative results and highlights the effectiveness of our architectural enhancements, especially the synergistic effect of channel attention for feature refinement and ASPP for multi-scale context integration.

To enhance model interpretability, Grad-CAM was applied to visualize the regions contributing to the model’s predictions. The resulting heatmaps, as shown in figure 2, indicate that the model consistently focuses on clinically relevant tumor regions rather than irrelevant background structures. This behavior suggests that the model learns meaningful feature representations aligned with tumor characteristics across different MRI modalities. The integration of attention mechanisms within the architecture further supports this interpretability by emphasizing informative feature channels during decoding. These observations provide additional evidence that the model’s predictions are not only accurate but also explainable, which is an important requirement for clinical adoption and trust in automated medical imaging systems.

The superior performance of our proposed method can be attributed to several factors. The channel attention mechanism helps the model focus on the most relevant features in each modality, while the ASPP module effectively captures multi-scale contextual information. When combined with the EfficientNet backbone, which provides a stronger feature extraction capability with fewer parameters, and data augmentation, which introduces anatomical variability and improves generalization, these components work synergistically to achieve more accurate and reliable segmentation results.

#### 4.3.2. Training convergence analysis

**Figure 3.**
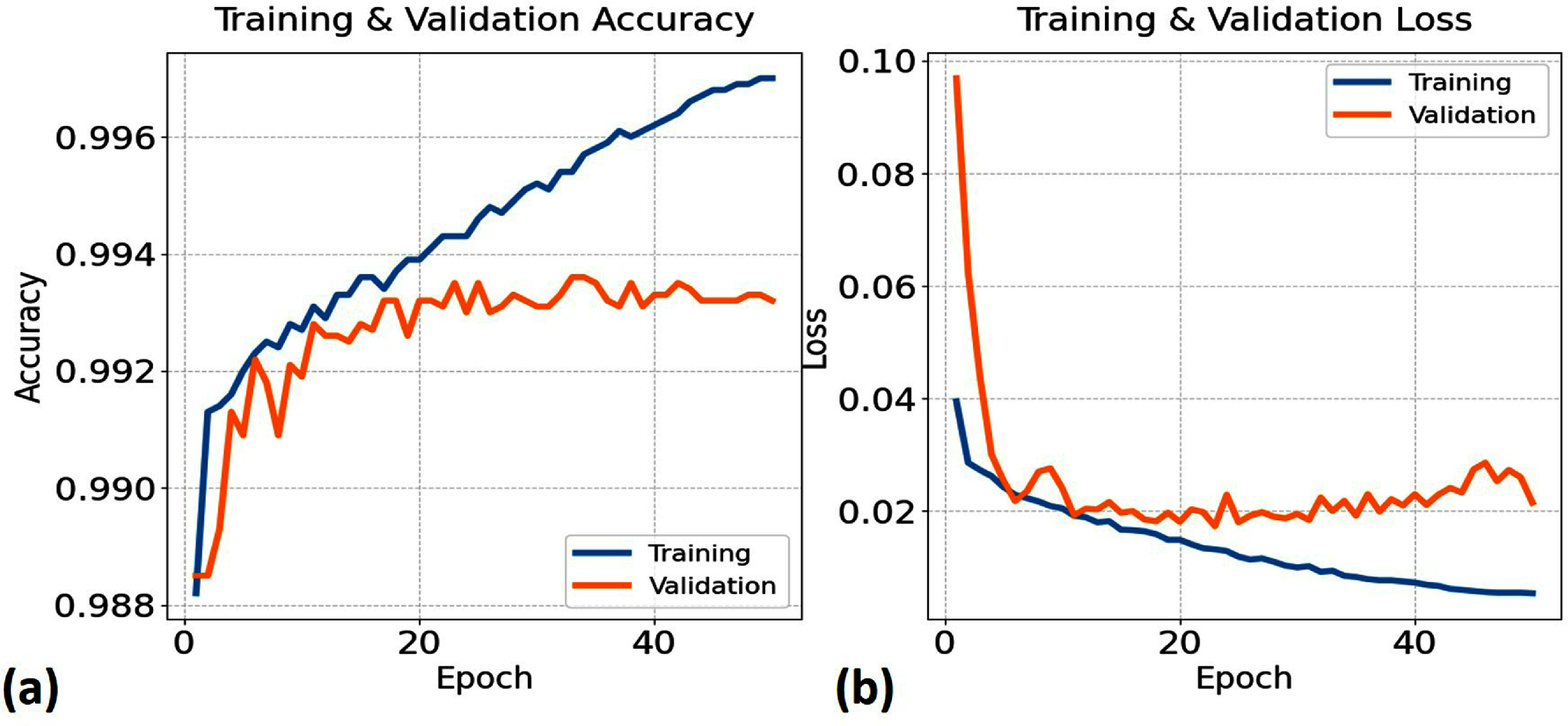
Training and validation accuracy and loss curves over 50 epochs, illustrating the convergence and stability of the model during optimization.

To examine the training behavior of the proposed model, the training and validation accuracy and loss curves are presented in figure 3. The curves show that the model converges within the early epochs and maintains stable performance throughout the remaining training process. The small gap between the training and validation curves suggests consistent generalization without evident overfitting. Additionally, the smooth and monotonic decrease in loss suggests effective convergence without oscillations or divergence, confirming the robustness of the training process.

#### 4.3.3. Comparison with the state-of-the-art methods

For a fair comparison, table 3 presents the performance metrics of our method against several state-of-the-art approaches on BraTS. The comparison includes both recent and established methods: ResU-net, nnU-Net, SCU-Net [46], Alternating time-space Transformer (ATST) [36], and Daza *et al* [47].

**Table 3.** Segmentation comparison of our method with the state-of-the-art.

	DSC			HD95		
Methods	ET	WT	TC	ET	WT	TC
ResU-net	0.722	0.851	0.767	7.20	11.65	5.54
nnU-Net	0.720	0.810	0.770	4.2	3.5	5.5
SCU-Net [46]	0.713	0.791	0.772	4.1	5.1	5.4
ATST [36]	0.785	0.9017	0.837	32.25	4.39	8.34
Cerberus [47]	0.794	0.897	0.845	29.82	3.59	6.47
Our method	**0.762**	**0.903**	**0.851**	**5.87**	**9.43**	**3.54**

Wang *et al*’s ATST method ranked second during the BraTS challenge by introducing a new method that relied on two interlinked pathways, each processing a pair of modalities [36]. Its method turned out to be quite efficient, particularly on whole tumor segmentation, where its DSC score was 0.9017. Similarly, Daza *et al* proposed a lightweight approach that showed the best performance in various metrics and resulted in the highest DSC values of 0.794, 0.897, and 0.845 for ET, WT, and TC, respectively [47].

Our method achieves competitive performance: 0.903 for whole tumor segmentation and 0.851 for TC segmentation, achieving a DSC of 0.762 for enhancing tumor, comparable to baseline methods such as ResU-net with 0.722 and nnU-Net at 0.720, though inferior compared to recent approaches like ATST and Daza *et al.* HD95 is rather inconsistent for the different tumor regions, with our method obtaining 5.87, 9.43, and 3.54 for ET, WT, and TC, respectively.

## 5. Conclusion

In this study, we proposed an integrated method based on ResU-Net for segmenting brain tumors by incorporating an EfficientNetB0 encoder, channel attention mechanism, and ASPP to reinforce the ability of multi-scale feature extraction and contextual learning. These demonstrated their performances on two benchmark datasets: TCGA LGG and BraTS 2020 and outperformed the baseline ResU-Net and its EfficientNet variant. The proposed approach shows the best performances in terms of DSC and HD95 scores on essential tumor regions, and it reaches a DSC of 0.903 and HD95 of 9.43 for the whole tumor on BraTS 2020. Compared with the state-of-the-art, our model exhibited competitive performance in whole tumor and TC segmentation, validating the effectiveness of combining a powerful encoder with an attention mechanism and ASPP for accurate boundary delineation. These results demonstrate that our approach demonstrates promising performance for clinical use, subject to further validation, and its further optimization and adaptation may be carried out on other medical segmentation tasks.

## Data Availability

The data that support the findings of this study are openly available at the following URL/DOI: https://github.com/majid9418/EResU-Net [48].
